# Editorial: Acquisition, processing, and maintenance of a new language: crosslinguistic perspectives on sequential bi/multilingualism

**DOI:** 10.3389/fnhum.2026.1964936

**Published:** 2026-09-04

**Authors:** Usha Lakshmanan, Isabelle Barrière

**Affiliations:** 1 Program in Brain and Cognitive Sciences, School of Psychological and Behavioral Sciences, Southern Illinois University Carbondale, Carbondale, IL, United States; 2 Speech-Language Pathology Program, Saint Elizabeth University and Research Institute for the Study of Language in Urban Society, City University of New York Graduate Center, New York, NY, United States

**Keywords:** cognitive neuroscience, crosslinguistic influence, heritage languages, language maintenance, language processing, neuroplasticity, second language acquisition, sequential/consecutive bilingualism

## Introduction

How the human brain adapts to a new language remains one of the most compelling questions in cognitive neuroscience. For decades, research has often relied on monolingual benchmarks, viewing an additional language as a secondary modification to a fixed native system (e.g., Blume et al., 2020). This Frontiers Research Topic explicitly challenges that static baseline. By compiling 14 original research contributions spanning various stages of life, this volume frames the multilingual mind as an active, constantly adapting network where multiple language subsystems continuously interact, compete, reorganize, and restructure. Using a diverse range of methodologies—including EEG, ERPs, real-time MRI, eye-tracking, naturalistic and experimentally elicited data, and sociolinguistic surveys—the contributors show how languages are acquired, processed in real time, and maintained or altered throughout a person's life. Their findings highlight these processes across a variety of frequently studied (e.g., Spanish-English) and under-studied (e.g., Turkish-Bulgarian, Kichwa-Spanish, Kiswahili-English) language combinations, within diverse cultural, demographic and national contexts.

## Theme 1: Neurobiological foundations and electrophysiological specialization

A primary concentration within this Research Topic leverages high-density neural tracking to evaluate early visual, auditory, and syntactic processing thresholds, highlighting the biological adaptation of the brain to new linguistic structures. Orthographic adaptation in logographic writing systems is explored by Hao et al., who show that advanced second language (L2) learners of Mandarin Chinese exhibit an automated, left-lateralized N170 wave response during character recognition tasks. This closely matches native speakers even when their first language (Indonesian) is alphabetic. However, persistent right-hemisphere activation suggests that native script constraints continue to shape early visual cortex tuning.

Auditory processing reveals a similar reliance on environmental input and specialized neural mapping. Bloder et al. analyze lateral temporal auditory evoked potentials (AEP) in Italian-German bilingual children, demonstrating that reduced exposure to German input systematically weakens the right temporal Ta-Tb response and decreases hemispheric differentiation for voice-onset-time stimuli. This highlights how sensitive early acoustic sensory commitment is to real-time, localized exposure profiles. At the level of complex grammar computation, Dekydtspotter et al. monitor event-related power differences (ERPDs) within gamma (γ) oscillations during live syntactic and semantic parsing. Their work uncovers a unified, dual-stage neurocomputational mechanism shared by native speakers of French and sequential learners of French alike: narrowband oscillations handle working-memory retrieval of referential elements before broadband outputs process hierarchical structural dependencies.

## Theme 2: Speech perception, production, and clinical/behavioral plasticity

Building upon these neurobiological layers, several contributors investigate how the bilingual system manages speech perception, sensorimotor mechanics, and clear speech targets under varied behavioral, spatial, or clinical conditions. Within a clinical framework, Kambhampaty et al. investigate central auditory tasks (CAT) in a large cohort of Kiswahili-speaking Tanzanian children managing chronic HIV infection. Their multivariate regression models offer a striking finding: active language learning can act as a helpful cognitive buffer. Children actively learning English L2 demonstrated significantly higher central auditory processing scores, independent of age or organic health constraints.

Sublexical speech decoding and the processing of phonetic acoustics are further examined by Wulfert et al., who weigh universal sonority principles against language-specific statistics during noise-embedded nonce word recognition in L1 and L2 German. Their results demonstrate that localized, real-world experience with consonant cluster distributions facilitates perception accuracy far more than abstract linguistic universals—a pattern particularly pronounced among L2 listeners. In the domain of motor production, Spinu et al. combine verbal memory spans with rtMRI to evaluate vocal tract kinematics during unfamiliar speech imitation, finding that high-proficiency Spanish-English bilinguals exhibited superior articulatory adaptation and enhanced working-memory primacy effects. Conversely, comparing Korean-English bilinguals living in the US and Korea for clear speech adjustments made to maintain acoustic clarity, Jung and Dmitrieva show that Korean speakers' long-term geographic immersion in an English L2 environment produces no significant variations in voice onset time (VOT) elongation, implying that adjustments for acoustic clarity are driven primarily by stable, internal target configurations rather than the dominant ambient language.

## Theme 3: Real-time parsing metrics vs. innate structural boundaries

The next thematic intersection captures the specific structural parameters and processing boundaries governing real-time online grammar parsing and referential processing. Pérez-Leroux et al. use natural speech eye-tracking to capture how adult Spanish-speaking bilinguals process phrasal gender marking in Spanish. Their tracking distributions show that while adult immigrants rapidly capitalize on determiner-level cues for real-time word prediction, heritage speakers of Spanish exhibit downstream processing delays when navigating morphophonologically alternating final vowels. Wang et al. track ambiguous pronoun resolution in Mandarin-English L2 learners. Their data expose a persistent processing bottleneck: although L2 learners successfully integrate discourse-level topicality cues into offline comprehension metrics, their real-time gaze movements default to L1 structural habits due to reduced online sensitivity to surface morphosyntactic information. Despite these online bottlenecks, the underlying resilience of innate grammar constraints remains robust. Bleotu et al. explore adjectival recursion under the Recursive Set-Subset Ordering (RSSO) constraint across adult sequential bilinguals with contrasting adjective word orders (post-nominal in L1 Romanian and pre-nominal in L2 English). Their behavioral outputs demonstrate that sequential learners seamlessly resolve recursive interpretations across both systems, validating the stability of deep Universal Grammar constraints against conflicting surface arrangements.

## Theme 4: Developmental, sociolinguistic, and methodological ecologies

The final theme moves into the broader field, examining how individual language development, social reasoning, and experimental setups interact with macro-level institutional architectures and policy domains. Focusing on internal grammar growth, Lust et al. track a young Korean child during an extended break from active English second-language input. Post-break testing documented significant syntactic gains and grammatical refinement despite zero environmental exposure to English. This surprising growth presents clear evidence for internal linguistic integration, showing that the mind continues to independently organize grammar even without active sensory data. Developmental intersections of social reasoning are further mapped by Kyuchuk, who traces Theory of Mind (ToM) milestones alongside evidentiality grammar in bilingual children in Bulgaria. The results suggest that Turkish-Bulgarian bilingual cohorts utilize alternative, non-verbal cognitive paths to pass social-reasoning tasks compared to Bulgarian monolinguals, whose success remains heavily anchored to explicit verbal structures.

Methodological frameworks and testing constraints within experimental pragmatics are evaluated by Gualapuro Gualapuro in a field study on quantity scalar implicatures among rural indigenous Kichwa-Spanish bilinguals in Ecuador. Focusing on real-world context, his work reveals that unfamiliar digital testing equipment creates artificial performance barriers. Simply replacing computer setups with familiar, offline materials facilitated participant accuracy scores to jump from 78% to 97%. This striking difference highlights the necessity of designing ecologically valid psycholinguistic experiments that consider real-world conditions and socioeconomic realities of local communities.

Finally, societal constraints that threaten language maintenance on a macro-structural scale are mapped by Sarma, who profiles over 1,000 university students to observe language shift within India's complex trilingual education hierarchy. Her tracking illustrates that global institutional pressures for a dominant global tongue (English) continuously alter individual dominance profiles, frequently shifting language dominance away from the mother-tongue (e.g., Hindi, and other regional Indic languages). This finding is somewhat consistent with other L1 attrition and L1 maintenance contexts. However, in work published elsewhere, Lakshmanan (2024) presents experimental evidence to support an alternative view on language maintenance within the same Indic context, demonstrating robust L1 Tamil maintenance among Tamil-English-Hindi multilinguals under specific conditions. Taken together, these contrasting findings highlight that traditional, migration-centric models of “heritage languages” are often too static to capture the fluid, multi-dialectal and multilingual shifts shaping modern societies.

## Conclusion and future trajectories

Synthesizing the 14 original contributions to this Research Topic demonstrates that managing multiple language systems involves a highly unified cognitive network. Changes or adaptations within one domain naturally trigger systemic adjustments across the broader linguistic architecture. By connecting precise electrophysiological and laboratory measures with descriptive field inventories, this volume provides a cohesive foundation for future work in human neuroscience, systemic language policy, and clinical linguistics across the lifespan.

Looking forward, the empirical findings point toward an integrated trajectory for future research. The discipline is increasingly moving away from static, single-language templates, advancing instead toward a dynamic approach that blends high-density, real-time neuroimaging with ecologically valid field-methods, inclusive of diverse communities and populations. Future research will likely prioritize three key areas: mapping fluid shifts in language dominance within complex, multilingual societies; exploring the therapeutic potential of language learning to build lifelong cognitive reserve; and delineating the precise boundaries where and how internal grammatical computations interact with external, real-world input.
